# METTL3 knockdown promotes temozolomide sensitivity of glioma stem cells via decreasing MGMT and APNG mRNA stability

**DOI:** 10.1038/s41420-023-01327-y

**Published:** 2023-01-23

**Authors:** Jia Shi, Peng Zhang, Xuchen Dong, Jiaqi Yuan, Yongdong Li, Suwen Li, Shan Cheng, Yifang Ping, Xingliang Dai, Jun Dong

**Affiliations:** 1 Department of Neurosurgery, Second Affiliated Hospital of Soochow University, Suzhou, China; 2 Department of Neurosurgery, Third Affiliated Hospital of Soochow University, Changzhou, China; 3 Department of Pathology and Southwest Cancer Center, Southwest Hospital, Third Military Medical University, Chongqing, China; 4 Department of Neurosurgery, First Affiliated Hospital of Anhui Medical University, Hefei, China

**Keywords:** CNS cancer, Cancer stem cells

## Abstract

Chemo-resistance hinders the therapeutic efficacy of temozolomide (TMZ) in treating glioblastoma multiforme (GBM). Recurrence of GBM even after combination of maximal tumor resection, concurrent radio-chemotherapy, and systemic TMZ applocation is inevitable and attributed to the high therapeutic resistance of glioma stem cells (GSCs), which can survive, evolve, and initiate tumor tissue remodeling, the underlying mechanisms of GSCs chemo-resistance, have not been fully elucidated up-to-now. Emerging evidence showed that METTL3-mediated N6-methyladenosine (m6A) modification contributed to the self-renew and radio-resistance in GSCs, however, its role on maintenance of TMZ resistance of GSCs has not been clarified and need further investigations. We found that the cell viability and half-maximal inhibitory concentration (IC50) of GSCs against TMZ significantly decreased after GSCs underwent serum-induced differentiation to adherent growth of tumor cells. Besides, METTL3 expression and total m6A modification declined dramatically in consistence with GSCs differentiation. Knockdown of METTL3 weakened self-renew, proliferation and TMZ IC50 of GSCs, whereas enhanced TMZ induced γH2AX level, indicating upregulation of double-strand DNA damage. We also found that mRNA stability of two critical DNA repair genes (MGMT and APNG) was regulated by METTL3-mediated m6A modification. In conclusion, we speculated that METTL3-mediated m6A modification of MGMT and APNG mRNAs played crucial roles on suppression of TMZ sensitivity of GSCs, which suggest a potential new therapeutic target of METTL3 against GBM.

## Introduction

Temozolomide (TMZ) was the first-line and one of the most commonly used chemo-therapeutic agent against glioblastoma multiforme (GBM) in clinical practice [1, 2]. In GBM patients, ubiquitous TMZ-resistance severely reduced the effectiveness of TMZ, causing tumor progression and recurrence, leading to poor prognosis [3]. Unraveling the underlying mechanisms for TMZ resistance was vital to solving this problem. TMZ resistance mechanisms were mainly associated with non-coding RNA regulatory network, DNA damage repair mechanisms, tumor microenvironment, abnormal signaling pathways and tumor cell autophagy, etc. [2, 4–8]. Several studies have suggested that GBM-derived glioma stem cells (GSCs) may play vital roles on formation of GBM resistance to TMZ [9–11]. GSCs had the features of self-renewal, infinite proliferation, multi-potential differentiation, strong infiltration, and migration abilities [11, 12]. It was well known that GSCs also showed high chemo-resistance [13]. However, the regulation mechanisms of TMZ sensitivity in GSCs largely remains unclear.

As early as the 1970s, scientists identified N6-methyladenosine (m6A) modification, which was a methylation modification capable of occurring on RNA adenine (A) such as mRNA, long non-coding RNA (lncRNA) [14]. Of the known post-transcriptional modifications of RNA, m6A was one of the most abundant modifications in most eukaryotic mRNAs and lncRNAs, accounting for 0.1–0.4% of adenosine and 50% of the total methylation of ribonucleotide in mammalian RNA [15]. Similar to epigenetic modifications such as DNA methylation and histone modification, m6A methylation was involved in the complex and fine biological regulation of important functional genes in various cellular processes [16, 17]. Emerging studies have shown that m6A methylation was involved in the occurrence of complex human diseases and especially played an important role on the occurrence and development of cancers [15, 18–20]. Current studies on m6A-related proteins have already shown that m6A methylation was a dynamically reversible processes, which was consisted of “Writers”, “Erasers”, and “Readers”. Writers refferred to a methylation enzyme editiing methylation modification into RNA, which was a modification process that mediated RNA methylation [21]. Writers had at least seven components to complete this process, including METTL3, METTL14, WTAP, RBM15, KIAA1429, HAKAI, and ZC3H13, etc. [22]. Among these regulating molecules, METTL3 and METTL14, responsible for catalyzing m6A methylation of mRNA [20, 23, 24], played important roles. METTL3-mediated m6A modification was reported to be critical in GSCs self-renew maintenance and radio-resistance [25]. Based on our previous studies, which disclosed that METTL3 promoted TMZ resistance in GBM cell lines via increasing MGMT (O6-methylguanine-DNA methyltransferase) and APNG (alkylpurine-DNA-N-glycosylase) in an m6A-dependent manner [26], but whether GSCs or their differentiated progeny tumor cells produced TMZ resistance in such a manner, has not been clearly charified up to now.

## Results

### GSCs showed weakened abilities of proliferation and TMZ resistance after differentiation

Two GSCs cell lines (GSC-11 and GSC-23) behaved typical neurosphere-like growth pattern with GSCs markers expression, including CD133, SOX2, Nestin, and CD44 by immunofluorescence assay (Supplementary Fig. 1). The proliferation ability of GSCs, indicated by EdU assay, decreased after serum-induced differentiation to an adhensive growth pattern (Supplementary Fig. 2A). Besides, after serum-induced differentiation, TMZ survival of differentiated GSCs, indicated by the TMZ IC50 value, decreased significantly from 2513 to 247.5 μM in GSC-11 cells, and 1565 to 85.15 μM in GSC-23 cells, respectively (Supplementary Fig. 2B).

### Total m6A RNA modification and METTL3 expression of GSCs were significantly higher than those of differentiated GSCs

**Fig. 1:**
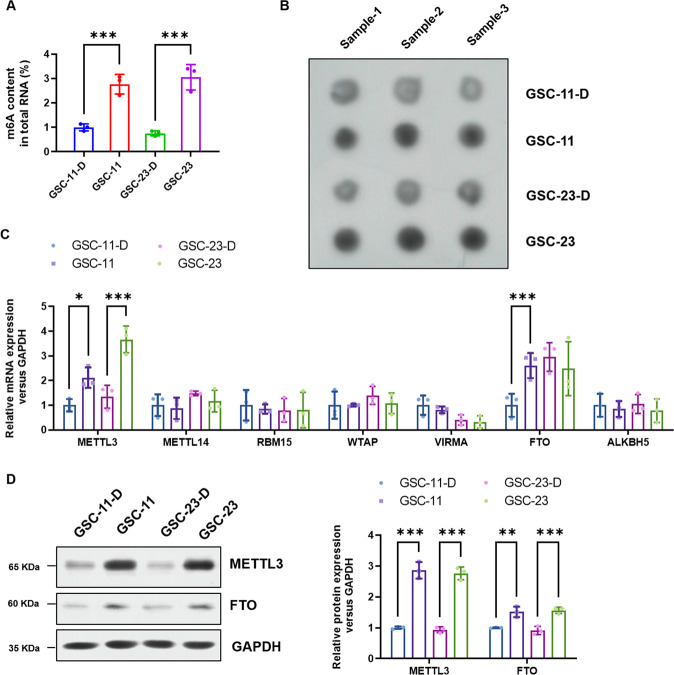
Both total m6A RNA modification and METTL3 expression of GSCs were significantly higher than those of the corresponding differentiated cells. **A** The colorimetric m6A quantification assay was conducted to examine the total m6A levels of two GSCs cell lines and the corresponding differentiated GSCs. **B** Dot Blot was performed to confirm the total m6A levels of two GSCs cell lines and differentiated GSCs. **C** The mRNA expression level of major m6A methyltransferases (METTL3, METTL14, RBM15, WTAP, and VIRMA) and demethylase (FTO and ALKBH5) of both two GSCs cell lines and their differentiated cells were analyzed by real-time PCR. **D** The dys-regulated level of METTL3 and FTO was confirmed by Western Blotting. *P ＜ 0.05, **P < 0.01 and ***P < 0.001 versus indicated groups.

To explore whether m6A RNA modification was involved in TMZ resistance maintenance of GSCs, total m6A RNA modification of GSCs was evaluated and compared with differentiated GSCs, which suggest the level of total m6A RNA modification of GSCs was significantly higher than that of differentiated GSCs (Fig. 1A), and were further verified by m6A Dot Blot (Fig. 1B). Besides, the expression level of major m6A methyltransferases (METTL3, METTL14, RBM15, WTAP, VIRMA) and demethylase (FTO, ALKBH5) were also analyzed, which showed that METTL3 mRNA level of GSCs was significantly higher than that of differentiated cells (Fig. 1C). The mRNA level of FTO in GSC-11 cells was higher than that in differentiated GSC-11 cells, whereas there was no obvious difference in FTO expression between GSC-23 and differentiated GSC-23 cells (Fig. 1C). The protein level of METTL3 and FTO in GSCs was significantly higher than that in their corresponding differentiated cells (Fig. 1D), upregulation of METTL3 in GSCs was obvious than that of FTO (Fig. 1D). Considering the consistency of of total m6A RNA modification and METTL3 in GSCs, we assumed that METTL3 overexpression contributed to m6A expression dysregulation of GSCs.

### METTL3 knockdown impaired self-renew and stemness maintenance of GSCs

**Fig. 2:**
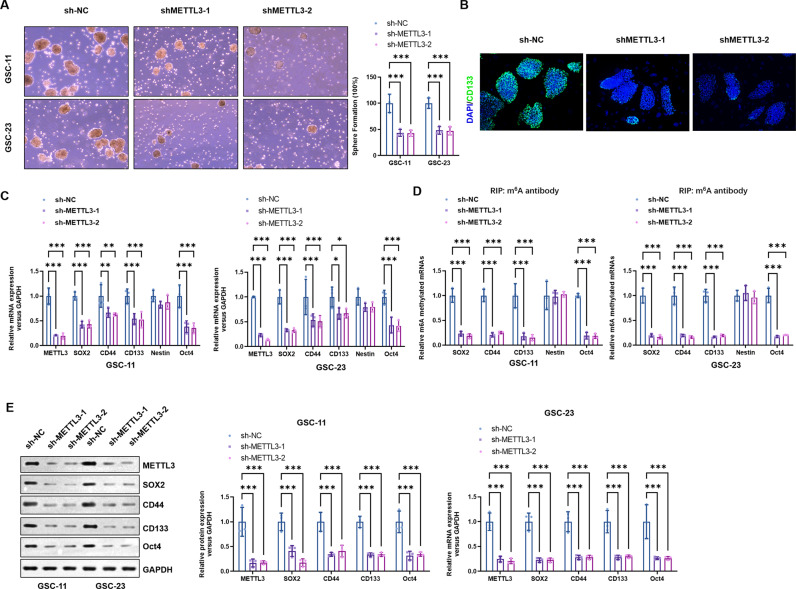
METTL3 knockdown impaired self-renew and stemness maintenance of GSCs. **A** METTL3 knockdown significantly decreased the number of tumorspheres formed by GSC-11 and GSC-23 cells in vitro. **B** IF analysis was performed to evaluate the effect of METTL3 knockdown on CD133 expression of GSC-11 cells. **C** Real-time PCR was performed to detect the effect of METTL3 knockdown on the mRNA levels of several stemness markers. **D** Me-RIP-PCR was applied to detect the m6A methylation levels of SOX2, CD44, CD133, Nestin and Oct4 in GSCs after METTL3 knockdown. **E** The effects of METTL3 knockdown on the protein levels of several GSCs markers were detected by Western blotting. *P < 0.05, **P < 0.01, ***P < 0.001 versus indicated groups.

To determine the functional effect of METTL3 on GSCs, METTL3 knockdown lentivirus vectors (shMETTL3-1 and shMETTL3-2) were constructed and transfected stably in GSCs. Tumorsphere formation ability of METTL3 knockdown GSCs were observed and compared with blank vector transfected GSCs, which showed that METTL3 knockdown significantly decreased tumorsphere number of both GSC-11 and GSC-23 cells (Fig. 2A). Besides, the influences of METTL3 knockdown on CD133 expression of GSCs was also investigated. Notably, immunofluorscence staining disclosed that METTL3 knockdown significantly diminished CD133 expression intensity in GSCs (Fig. 2B). Furthermore, METTL3 knockdown comparably decreased transcriptional (Fig. 2C) and expression (Fig. 2E) levels of GSCs markers, including SOX2, CD44, CD133 and Oct4, however, glial precursor cell marker Nestin expression had not been affected with METTL3 knockdown in GSCs. We further detected the m6A methylated transcripts in the GSCs and found that METTL3 knockdown decreased the m6A methylated mRNAs of SOX2, CD44, CD133, and Oct4 (Fig. 2D). These results demonstrated that METTL3 knockdown impaired self-renew and stemness maintenance of GSCs, which might be related to the decreased m6A modification of transcripts.

### METTL3 knockdown improved TMZ sensitivity of GSCs and elevated TMZ-induced DNA damage

**Fig. 3:**
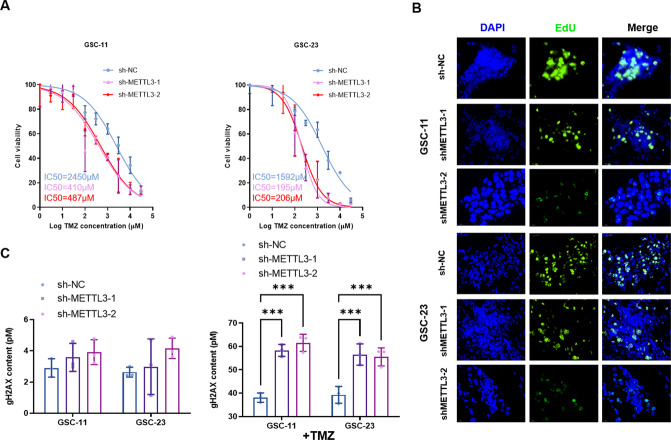
METTL3 knockdown in GSCs not only improved TMZ sensitivity of GSCs, but also elevated TMZ-induced DNA damage. **A** The effect of METTL3 knockdown (shMETTL3-1 and shMETTL3-2) on TMZ IC50 value of GSCs (GSC-11 and GSC-23) was analyzed by CCK-8 assay. **B** Edu assay was applied to evaluate the effect of METTL3 knockdown (shMETTL3-1 and shMETTL3-2) on proliferation ability of GSCs (GSC-11 and GSC-23). **C** The level of γH2AX content was analyzed with ELISA to evaluate DNA damage extent of GSCs (GSC-11 and GSC-23) after TMZ administration. ***P < 0.001 versus indicated groups.

To explore the potential role of METTL3 on TMZ sensitivity regulation in GSCs, METTL3-kockdown GSCs were cultured in vitro with addition of TMZ. As the results showed, the TMZ IC50 values of GSCs after METTL3 knockdown significantly decreased from 2450 μM (GSCs-11)/1592 μM (GSCs-23) to 410 (GSCs-11-shMETTL3-1) or 487 (GSCs-11-shMETTL3-2)/195 μM (GSCs-23-shMETTL3-1) or 206 μM (GSCs-23-shMETTL3-2), respectively (Fig. 3A). Consistently, proliferation ability of GSCs, evaluated by EdU assay, decreased obviously after METTL3 knockdown (Fig. 3B). DNA damage level of GSCs, indicated by ©H2AX content, increased significantly after METTL3 knockdown under TMZ exposure (Fig. 3C). These results indicated that METTL3 knockdown imporved TMZ sensitivity of GSCs and enhanced TMZ-induced DNA damage.

### METTL3 knockdown decreased mRNA stability of two critical DNA repair genes (MGMT and APNG)

**Fig. 4:**
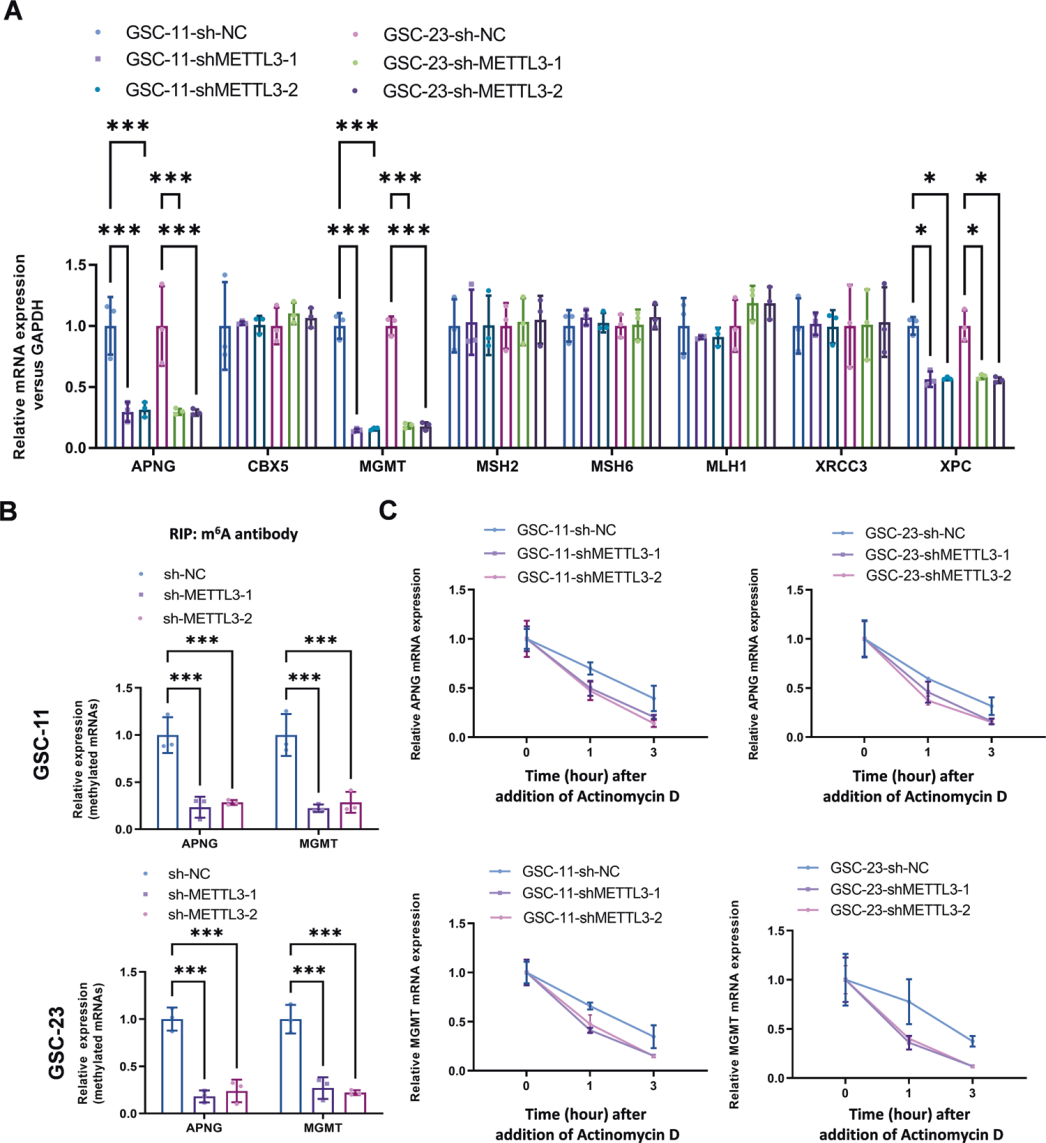
METTL3 knockdown downregulated mRNA stability of two critical DNA repair genes (MGMT and APNG) in GSCs. **A** The effect of METTL3 knockdown (shMETTL3-1 and shMETTL3-2) in GSCs (GSC-11 and GSC-23) on the level of major repair enzymes, including APNG, CBX5, MGMT, MSH2, MSH6, MLH1, XRCC3, and XPC, was analyzed by real-time PCR. **B** The effect of METTL3 knockdown (shMETTL3-1 and shMETTL3-2) on m6A methylated level of APNG and MGMT was analyzed by Me-RIP-qPCR. **C** The effect of METTL3 knockdown (shMETTL3-1 and shMETTL3-2) on mRNA stability of APNG and MGMT was evaluated by transcription assay *P < 0.05 and ***P < 0.001 versus indicated groups.

To explore whether METTL3 alteration affect the level of major DNA repair enzymes, the expression status of APNG, CBX5, MGMT, MSH2, MSH6, MLH1, XRCC3, and XPC were detected in METTL3 knockdown GSCs (shMETTL3-1 and shMETTL3-2), which showed METTL3 knockdown significantly decreased the expression level of APNG and MGMT in both GSC-11 and GSC-23 cells (Fig. 4A) m6A methylated level of APNG and MGMT significantly decreased in METTL3 knockdown GSC-11 and GSC-23 cells (Fig. 4B), and mRNA stability of APNG and MGMT, evaluated by the corresponding transcriptional assay, decreased similarly in METTL3 knockdown GSC-11 and GSC-23 cells (Fig. 4C). Taken together, these results suggest that METTL3 knockdown impaired mRNA stability of two critical DNA repair genes MGMT and APNG.

### METTL3 knockdown improved the TMZ sensitivity of GSCs in vivo

**Fig. 5:**
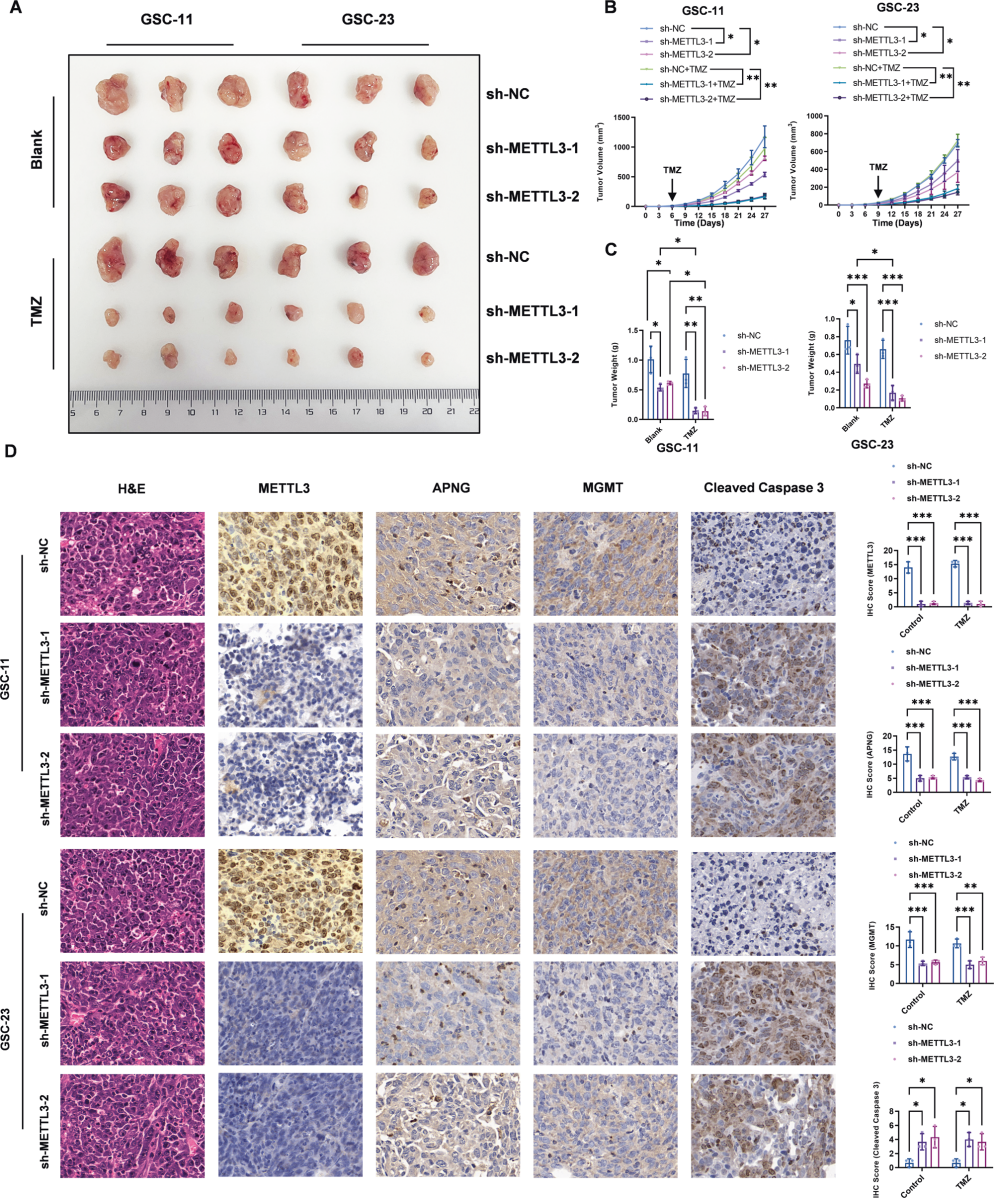
In vivo tumorigenicity assay disclosed METTL3 knockdown improved TMZ sensitivity of GSCs. **A** The xenograft tumor size of NC or METTL3 knockdown GSC-11 and GSC23 cells with or without TMZ treatment. **B** Tumor growth curve. **C** Weight of the implanted tumors from GSCs in subcutaneous xenograft model of BALB/c nude mice with or without TMZ treatment. **D** IHC analysis of METTL3, MGMT, APNG, and cleaved caspase-3 in subcutaneous model. *P ＜ 0.05, **P ＜ 0.01, ***P < 0.001 versus indicated groups.

To investigate whether METTL3-mediated m6A modification contributes to TMZ sensitivity regulation of GSCs in vivo, subcutaneous inoculation of 5 × 10^6^ control or METTL3 knockdown GSC-11/GSC-23 cells were performed on BALB/c nude mice. After subcutaneous xenograft reached about 100 mm^3^, mice were treated with or without TMZ (100 mg/kg/d, 5 days) with intragastric administration. TMZ did not affect the tumor weight and tumor volume in sh-NC GSC-11/GSC-23 cells implanted mice (Fig. 5A–C). The obvious difference in both weight and volume of xenografts was observed between METTL3 knockdown GSC-11/GSC-23 and control GSC-11/GSC-23 cells implanted mice (Fig. 5A–C). METTL3 knockdown GSC-11/GSC23 cells formed xenografts were significantly smaller than those of GSC-11/GSC23-NC cells. IHC staining was further performed to evaluate the expression levels of METTL3, APNG, MGMT, and cleaved Caspase 3, which showed that the xenograft tumors generated from METTL3 knockdown GSC-11/GSC-23 cells expressed lower level of APNG and MGMT, and higher level of cleaved Caspase 3 (Fig. 5D, E). Collectively, these results implied that METTL3 knockdown improved TMZ sensitivity of GSCs in vivo.

## Discussion

MGMT is a DNA repair enzyme with variable expression in GBM. High expression level of MGMT in GBM is associated with significant resistance to TMZ [27]. TMZ can induce methylation of O6-MG on DNA, and MGMT can transfer O6-MG admixture caused by TMZ to its cysteine residue to avoid DNA crosslinking and breaking, so as to repair DNA of tumor cells, thus resulting in drug resistance of GBM to alkylating agent, and irreversible inactivation of its own, namely “suicide” repair mechanism [28].

APNG, as base excision repair enzyme, repairs the cytotoxic lesions of N3-methyladenine and N7-methylguanine, which is also critical to TMZ resistance maintenance [29]. We previously demonstrated that METTL3-mediated m6A modification contributed to TMZ resistance in GBM cell lines [26]. However, there were various heterogeneous cell populations in GBM cells, including GSCs with stemness and natural chemotherapeutic tolerance. Therefore, to clarify the relationship between GSCs chemoresistance and METTL3-mediated m6A modification, we detected the expression of METTL3 and m6A modification in both GSCs and corresponding differentiated tumor cells and confirmed that METTL3 promoted GSCs resistance to TMZ via increasing MGMT and APNG in a m6A-dependent manner.

High chemotherapeutic resistance is one of the representative characteristics of GSCs, which contributed mainly in chemotherapeutic resistance formation in GBM [9]. TMZ is the first-line chemotherapeutic agent with definite clinical effect against GBM, however, drug resistance of GSCs limited its clinical efficacy [5]. MGMT has been reported to be the core mechanism of TMZ resistance in gliomas. Patients with unmethylated or overexpressed MGMT had strong resistance to TMZ and poor overall survival [30]. The mechanisms related to TMZ resistance also referred to autophagy and hypoxic microenvironment of GBM [31, 32], but high chemotherapeutic resistance of GSCs can not be reversed.

Recently, several innovative pre-clinical studies have emerged to enhance tumor inhibiting efficacy of TMZ [4, 7, 33]. Different treatment strategies based on various mechanisms of TMZ resistance can reduce GBM resistance to TMZ to some extent both in vitro and in vivo, and may offer new hope to improve GBM patients prognosis [8, 11, 12, 34]. More studies have well demonstrated that GSCs were more resistant to TMZ than their differentiated progeny cells [35]. However, METTL3-mediated m6A modification in maintenance of TMZ resistance of GSCs has not been fully investigated previously.

Translations studies on the relationship between m6A mRNA methylation and tumor chemotherapeutic resistance have potential significance in guiding clinical practice [36], for m6A mRNA methylation has been reported to be closely related to occurrence and development of tumors [37], and m6A-related protein has been proved to be an important regulatory factor in occurrence and development of tumors, besides, its expression level was associated with pathological grades of cancer [38]. However, in different malignancies, m6A modification can play either oncogenic or tumor-suppressive roles [39, 40]. Therefore, it is of great significance to clarify the biological functions of m6A-regulated genes in each malignancy, and identify key m6A target genes for understanding the pathogenesis of cancer as well. Recent studies have found that GSCs were closely related to m6A modification [41]. Visvanathan et al. first explored the methylation of m6A RNA in GSCs, and found that m6A methylation was reduced during GSCs differentiation in vitro. Moreover, high expression of METTL3 in GSCs increased the stability of SOX2 mRNA, and contributed to radio-resistance [25, 42], suggesting that METTL3 may play an important role in the pregression of chemoresistance. Besides that, currently, temozolomide resistance strongly limit the potential targets of GBM and novel mechanisms underlying drug-fast are urgently needed. Previous studies indicated that TMZ intervention upregulated METTL3, which induced the m6A modification of histone modifiers. Depletion of METTL3 promoted nonsense-mediated mRNA decay (NMD) of histone modifiers, thereby affecting the features of GSCs [43]. Study by Li et al. found that m6A methyltransferase METTL3 served as a NMD regulator of splicing factors. Knockdown of METTL3 suppressed the self-renewal of GSCs [44]. However, gap between METTL3 and TMZ resistance, especially the potential mechanisms and regulatory modes of DNA damage repair genes, need to be further explored. In this study, results showed that both mRNA m6A methylation and the expression level of METTL3 in GSCs were significantly higher than that of differentiated GSCs. Interestingly, unlike the protein level, the mRNA level of FTO in GSC-11 cells was significantly higher than that of differentiated cells, whereas FTO level in GSC-23 cells was similar to differentiated GSC-23 cells, which implied that FTO upregulation may be a feedback of elevated m6A methylation. By DNA damage repair genes screening, APNG and MGMT were identified as downstream of METTL3. Me-RIP-PCR and mRNA stability assay confirmed the transcriptional regulation of METTL3 on target genes.

On one hand, inhibition of METTL3 or METTL14 expression can reduce m6A level, enhance GSCs growth and self-renewal in vitro, as well as promote tumor-forming ability of GSCs in vivo [41, 45]. On the other hand, METTL3 overexpression or FTO inhibitor (Meclofenamic acid 2, MA2) can increase m6A level of GSCs, thus inhibit GSCs growth and slow down occurrence and development of GBM [45]. Furthermore, in PBT003 GBM cell line, METTL3 or METTL14 knockdown led to upregulation of several oncogenes (e.g., ADAM19, EPHA3, and KLF 4) and downregulation of tumor suppressors (e.g., CDKN2A, BRCA2, and TP53I11), conversely, overexpression of METTL3 or treatment with FTO inhibitor MA2 resulted in reduced expression of these oncogenes [45]. Another study showed that m6A demethylase ALKBH5 highly expressed in GSCs, and inhibition of ALHKBH5 reduced self-renewal, proliferation, and tumorigenesis of GSCs [46]. ALKBH5 demethylated a new transcripts FOXM1 (a key transcription factor in cell cycle regulation and plays a key role in GSCs self-renewal and tumorigenesis) and enhanced FOXM1 expression by binding to 3’UTR [46]. The nuclear lncRNAFOXM1AS (antisense of nuclear lncRNA) of FOXM1 can further promote the interaction between FOXM1 newborn transcript and ALKBH5 to enhance this process [46]. Knockout of FOXM1-AS also destroyed FOXM1 expression and self-renewal in GSCs, and overexpression of FOXM1 can rescue tumor growth of GSCs after depletion of ALKBH5 or FoxM1-AS, which further disclosed the key role of FOXM1 in GSCs initiated tumorigenesis [46]. Our data disclosed that METTL3 knockdown comparably decreased the mRNA and protein levels of several GSCs self-renewal associated factors, including SOX2, CD44, CD133, Nestin, and Oct4, etc. Moreover, METTL3 knockdown diminished stemness maintenance and self-renew of GSCs. Therefore, m6A methylation and demethylation on GSCs may affect GBM development through different regulatory mechanisms, thus playing a key role in the occurrence and development of GBM and providing a new therapeutic target against such refractory malignancy.

The current study suggests that maintenance of TMZ resistance in GSCs was regulated mainly by the METTL3-mediated m6A modification, and downregulation Knockdown of METTL3 dramatically impaired the TMZ resistance and aggravated TMZ-induced DNA damage in GSCs, which confirmed that METTL3 knockdown obviously decreased mRNA stability and improved TMZ sensitivity in GSCs.

Collectively, our study showed that METTL3 played a crucial role in regulation of TMZ sensitivity via regulating the mRNA stabilities of MGMT and APNG mRNAs in GSCs, which may serve as the potential treatment strategies targeting METTL3 to reverse TMZ resistance against GBM.

## Materials and methods

### Cell line and cell culture

Patient-derived human GSC-11 and GSC-23 (MD Anderson Cancer Center, University of Texas) were cultured favored for tumorsphere growth in DMEM/F12 medium supplemented with B27 supplement (Gibco, Grand Island, NY, USA), bFGF (Gibco, 20 ng/mL), and EGF (Gibco, 20 ng/mL) in an incubator (Sanyo, Japan) with humidified atmosphere at 37 °C with 5% CO_2_. For GSCs differentiation-inducing culture, GSC-11 or GSC-23 cells were seeded in culture flask in DMEM with 10% FBS (Gibco) and 1% penicillin-streptomycin (Gibco) for 1 week.

### Immunofluorescence (IF) staining

A total of 1 × 10^4^ GSCs per well of 24-well plates were fixed with 4% formaldehyde (Sangon Biotech, Shanghai, China). After blocking with normal goat serum (5%, Sangon Biotech, Shanghai, China) and permeabilizing with 0.1% Triton X-100, IF was performed using the primary antibodies (anti-CD133, anti-SOX2, anti-Nestin, or anti-CD44, respectively, dilution 1:400) and the secondary antibody Alexa Fluor® 594 (Thermo Fisher, dilution 1:1000). Nuclei were counterstained with DAPI. Images were captured under Zeiss LSM800 confocal imaging system.

### Cell viability assay

Cell viability of GSCs (1000 cells/well) and the corresponding serum-induced differentiated progeny tumor cells were measured after treatment with TMZ (Selleck Chemicals, Houston, TX, USA) at different concentrations (0–30,000 μM). After subsequent routine culture for 24 h, 10 μL CCK-8 reagent (Sangon Biotech) was added, and OD450 value was detected with ultra-multifunctional microplate analyzer (Tecan, USA). The “Log (inhibitor) vs normalized slope of response variable” method was applied with GraphPad Prism 9.0 to calculate the half-maximal inhibitory concentration (IC50) of TMZ.

### EdU assay

5-Ethynyl-2′-deoxyuridine (EdU) assay was performed using an EdU detection kit (Genelily, Shanghai, China). Briefly, a total of 1 × 10^4^ GSCs per well of 24-well plates were incubated in medium favored for tumor-sphere growth containing EdU (50 μM) for 24 h in humidifying atmosphere at 37 °C and 5% CO_2_. Subsequently, GSCs were fixed with 4% formaldehyde (Sangon Biotech) for 20 mins and permeabilized with 0.5% Triton X-100 for 5 min, then were washed with PBS and stained with 1× staining solution at room temperature for 30 min. Nuclei were counterstained with DAPI. Images were taken with Zeiss LSM800 confocal imaging system.

### Total RNA m6A quantification

Total m6A level in GSCs (1 × 10^4^ GSCs per well of 24-well plates) was analyzed using the EpiQuik™ m6A RNA Methylation Quantitative Kit (Epigentek, USA). Briefly, total RNA was isolated with Trizol reagent (Thermofisher, USA) according to the manufactuer’s method. RNA was added to each well (200 ng/well) of the 96-well plate from the quantitative kit, followed with a mixture of capture antibodies and detection antibodies incubation for 0.5 h. Then m6A content was quantified at 450 nm and calculated according to the standard curve following the manufacturer’s instruction.

### Real-time quantitative PCR (qRT-PCR)

Total RNA was isolated with Trizol reagent (Thermofisher, USA) according to the manufactuer’s method. The cDNA was synthesized using ReverTra Ace™ qPCR RT Kit (TOYOBO, Japan). The quantitative real-time fluorescent quantitative PCR (qRT-PCR) procedures were performed using the ABI PRISM 7900 HT Sequence Detection System (Life Technologies, USA). The relative gene expression of mRNA was calculated by the 2^-ΔΔCT^ method. GAPDH was used as an internal control to normalize the data. The primer information used in this study was in Supplementary Table 1.

### m6A dot blot

m6A dot blot was performed as previously described [47]. Briefly, after spotting with RNA samples (400 ng RNA/Dot), the nylon membrane (GE Healthcare, UK) was UV cross-linked and blocked for 1 h in 5% fat-free dry milk in 0.1% PBST, then incubated with the specific m6A antibody (1:1000, Abcam) overnight at 4 °C. Following extensive washing with 0.1% PBST, HRP-conjugated anti-rabbit IgG was diluted 1:5000 in Blocking Buffer and added to the membrane for 1 h at 25 °C, then the membrane was visualized with Bio-Rad imaging system.

### Western blotting

After lysed with RIPA buffer and the protein sample (20 μg/band) were separated with 1×SDS-PAGE, protein bands transferred into the PVDF membrane (Merck Millipore) were detected with the primary antibodies (anti-METTL3 antibody (1:1000), anti-FTO antibody (1:1500, Abcam) first, then HRP-bound secondary antibody. The membrane was visualized with Chemiluminescence Detection Kit (Servicebio, China) and detected under a Bio-Rad imaging system (Bio-Rad, USA).

### Methylated RNA immunoprecipitation (Me-RIP)-qPCR

5 µl anti-m6A antibody (Abcam) was coupled to Protein A agarose beads (Sigma-Aldrich, USA) in 200 µl of 1 M immunoprecipitation (IP) Buffer for 4 h at 4 °C. Then beads were washed 3 times in 200 µl of 140 mM IP Buffer. A total of 10 μg RNA was denatured by keeping at 75 °C for 5 min, cooled on ice for 2–3 min, and bound to antibody-coupled beads in 200 µl of 140 mM IP Buffer. Beads were treated with 100 µl Elution Buffer for 2 h at 50 °C, and RNA was recovered with phenol: chloroform extraction followed by ethanol precipitation. The level of MGMT and APNG in RNA were assayed by qRT-PCR.

### RNA stability assay

After 24 h of plating, Actinomycin D (5 μg/ml, Selleck) was added to GSCs (1 × 10^4^ GSCs per well of 24-well plates). After incubation for 1–3 h, cells were collected and RNA was extracted for further qRT-PCR. The mRNA’s half-life (t1∕2) was calculated using ln2∕ slope, and GAPDH was used for normalization.

### GSCs xenograft model

All xenograft experiments involving mice were conducted following the Ethical Standards of the Animal Care and Use Committee of Soochow University. To establish the xenograft model of GSCs in mice, GSCs or METTL3 knocking-down GSCs (v/v = 1:1) were subcutaneously inoculated into the right posterior limb of BALB/c nude mice (5 × 10^6^ cells/mice, 4-week-old, female, body weight of 20 g). Tumor volume was measured with calipers every 3 days. The tumor size was calculated using the formula: (*a*^2^ × *b*)/2 (*a*: width in mm, *b*: length in mm). After about 27 days, all mice were sacrificed under general anesthesia and the xenografts were harvested for further pathological study.

### Statistical analysis

Statistical analysis was performed using GraphPad Prism 9.0 (GraphPad Software, San Diego, CA, USA). The data in all figures are expressed as mean ± SEM. Student *t*-test, one-way analysis of variance (ANOVA), and two-way ANOVA were used to compare differences between and among groups. For all statistical tests, *P* < 0.05 (bilateral) was considered statistically significant.

## Supplementary information

Supplementary Figure legends

Supplementary Figure 1

Supplementary Figure 2

Supplementary Table 1

Original Data File

Original Data File

## Data Availability

All data were generated or analyzed during this study are available from the corresponding author on reasonable request.
