# Supplementary material for: METTL3 knockdown promotes temozolomide sensitivity of glioma stem cells via decreasing MGMT and APNG mRNA stability
Source: Cell Death Discov. 2023 Jan 23;9:22. doi: 10.1038/s41420-023-01327-y (PMC9868123; doi:10.1038/s41420-023-01327-y)
Supplement: Supplementary file 1 — Supplementary Figure legends [file 41420_2023_1327_MOESM1_ESM.docx]

**Supplementary Figure 1.** **Expression of GSCs markers**. CD133, SOX2, nestin, and CD44 in patient-derived GSCs (GSC-11 and GSC-23) were verified by immunofluorescence.

**Supplementary Figure 2.** **TMZ resistance and proliferation ability of GSCs were higher than those of differentiated GSCs.** (A) Edu assay was performed to evaluate proliferation ability of GSCs and their differentiated GSCs. (B) The TMZ IC50 value of GSCs (GSC-11, GSC-23) and their corresponding differentiated GSCs (GSC-11-D, GSC-23-D) were analyzed with CCK-8 assay.
