# Supplementary figures and images for: METTL3 knockdown promotes temozolomide sensitivity of glioma stem cells via decreasing MGMT and APNG mRNA stability
Source: Cell Death Discov. 2023 Jan 23;9:22. doi: 10.1038/s41420-023-01327-y (PMC9868123; doi:10.1038/s41420-023-01327-y)

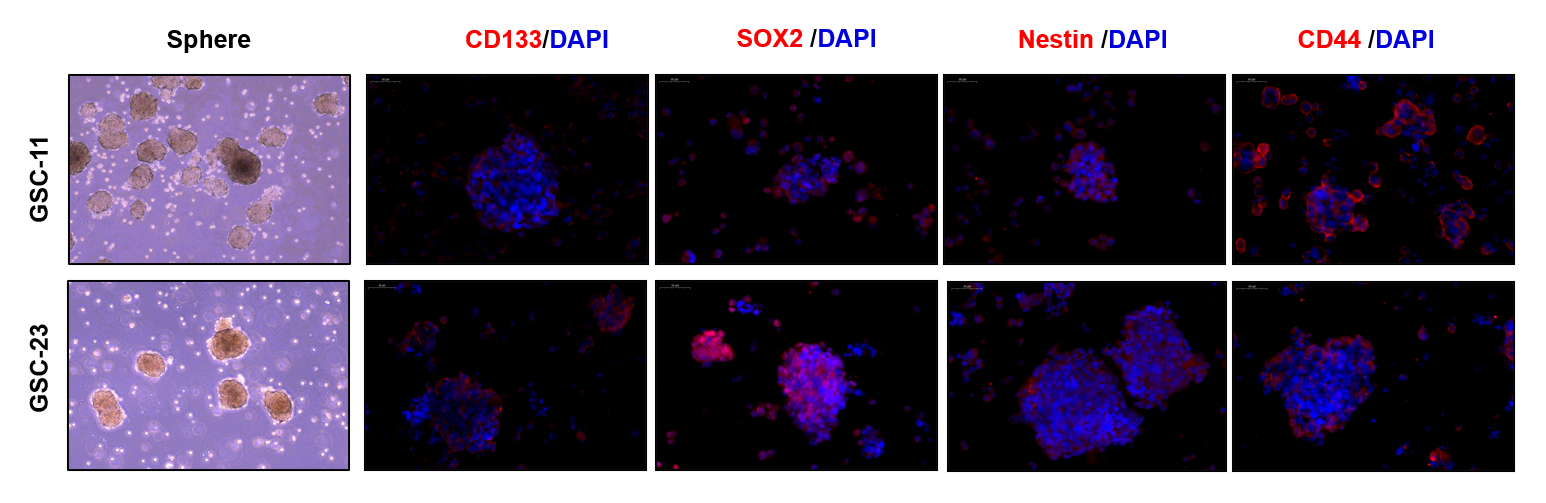

Supplement: Supplementary file 2 — Supplementary Figure 1 [file 41420_2023_1327_MOESM2_ESM.tif]

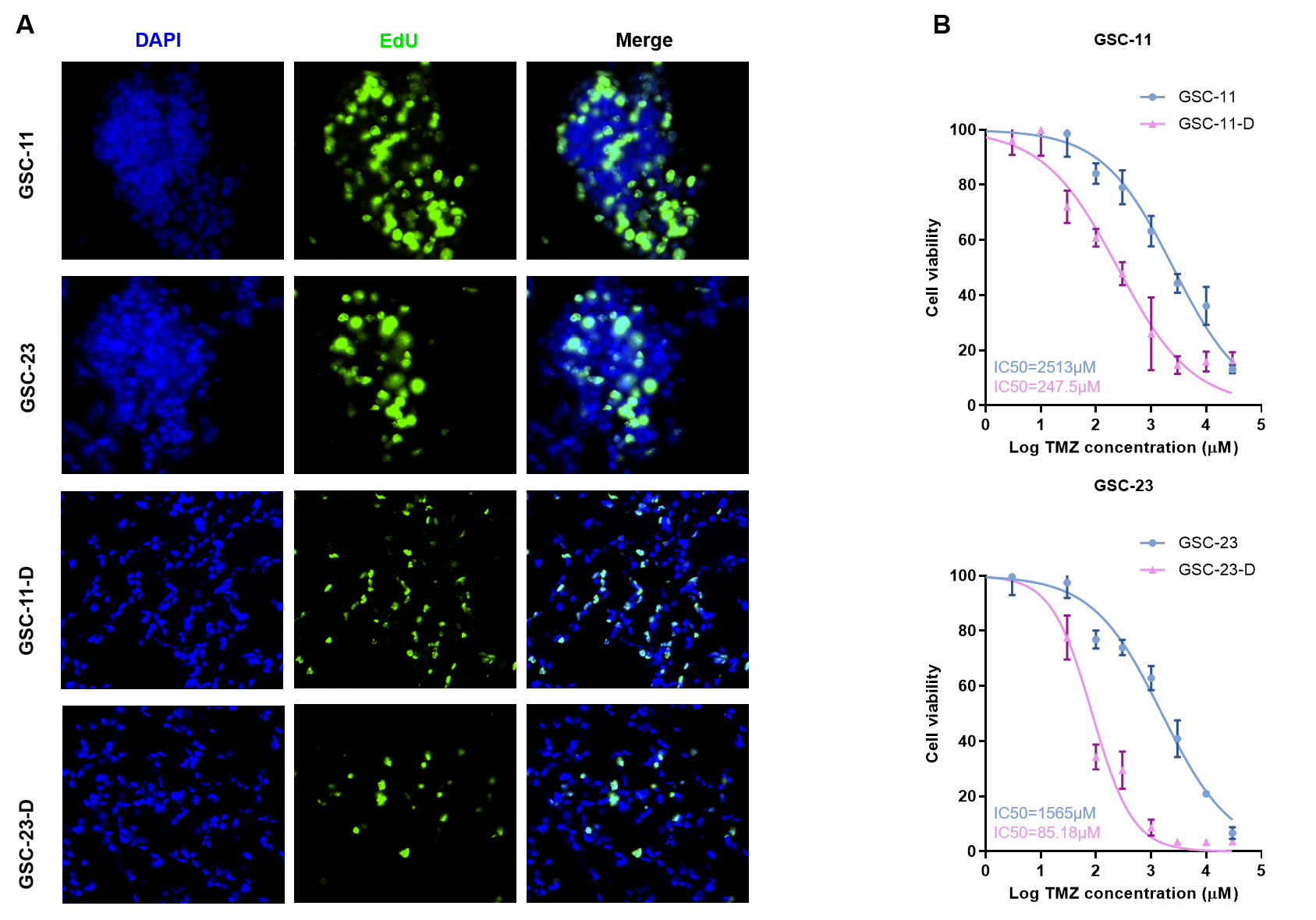

Supplement: Supplementary file 3 — Supplementary Figure 2 [file 41420_2023_1327_MOESM3_ESM.tif]

## Slide 1
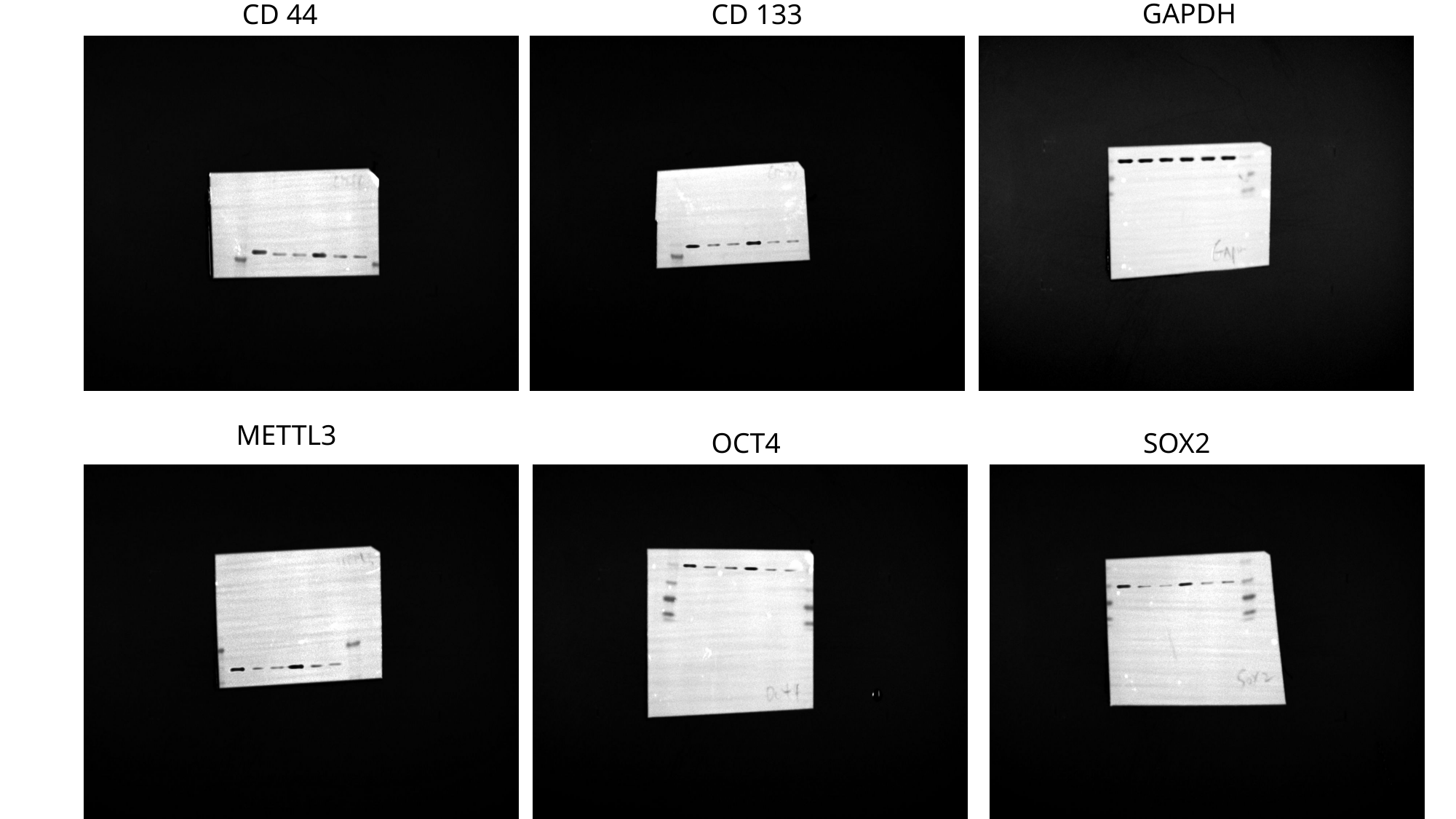

GAPDH
CD 44
CD 133
METTL3
OCT4
SOX2

Supplement: Supplementary file 6 — Original Data File [file 41420_2023_1327_MOESM6_ESM.pptx]
