# Supplementary material for: METTL3 knockdown promotes temozolomide sensitivity of glioma stem cells via decreasing MGMT and APNG mRNA stability
Source: Cell Death Discov. 2023 Jan 23;9:22. doi: 10.1038/s41420-023-01327-y (PMC9868123; doi:10.1038/s41420-023-01327-y)
Supplement: Supplementary file 4 — Supplementary Table 1 [file 41420_2023_1327_MOESM4_ESM.docx]

| **Genes** | **Forward (5’-3’)** | **Reverse (5’-3’)** |
| --- | --- | --- |
| METTL3 | CAAGCTGCACTTCAGACGAA | GCTTGGCGTGTGGTCTTT |
| METTL14 | CTGGGGAGGGGTTGGACCTT | CCCCGTCTGTGCTACGCTTC |
| RBM15 | TCCCACCTTGTGAGTTCTCC | GTCAGCGCCAAGTTTTCTCT |
| WTAP | CTTCCCAAGAAGGTTCGATTGA | TCAGACTCTCTTAGGCCAGTTAC |
| VIRMA | AATCCTGTGGGAAGATCAGC | ACACGTAAGGCAGTGGTAAG |
| FTO | CCAGAACCTGAGGAGAGAATGG | CGATGTCTGTGAGGTCAAACGG |
| ALKBH5 | CCAGCTATGCTTCAGATCGCCT | GGTTCTCTTCCTTGTCCATCTCC |
| APNG | AAGGGCCACCTTACCCGACT | GAGTGGGCGGCTTCATCCTC |
| CBX5 | GGCGCGTGGTTAAGGGACAA | CCTCTCTCAAAGCCCCGAGC |
| MGMT | CACTGGACAGCCCTTTGGGG | ATAGCCTCGGGCTGGTGGAA |
| MSH2 | AGACGCTGCAGTTGGAGAGC | GCCGTGCGCCGTATAGAAGT |
| MSH6 | GAGATCGGTAGCGCCTGCTG | GCCCCTTGTTGGGCTGTCAT |
| MLH1 | GTTATTCGGCGGCTGGACGA | GGCCAAAGCCTCACCTCGAA |
| XRCC3 | CGCCTGGTGGTCATCGACTC | ACCAGGAGCTGGTTAGCCCA |
| XPC | AGACTGTGTGCACGGTGTGG | GTGGGCAAAGGCTGGTCCAT |
| SOX2 | TACATGAACGGCTCGCCCAC | GCCCTGGAGTGGGAGGAAGA |
| CD44 | CGTGATGGCACCCGCTATGT | GTGCTGTCGGTGATCCAGGG |
| CD133 | GGAGGGCAGCCTTCATCCAC | AAACAGCAGCCCCAGGACAC |
| Nextin | TCACCCTTGCCTGCTACCCT | TCCTCCCACCCTGTGTCTGG |
| Oct4 | AGCAGATCAGCCACATCGCC | AAGGAGACCCAGCAGCCTCA |
| GAPDH | GCGGGGCTCTCCAGAACATC | TCCACCACTGACACGTTGGC |

**Supplementary Table 1.** The sequences of the primers.
